# Rotation-driven changes in physicochemical properties modulate soil microbial diversity and community complexity in tobacco-woad soils

**DOI:** 10.1128/spectrum.03016-24

**Published:** 2025-11-17

**Authors:** Zhongyan Wang, Xiaomeng Guo, Hongfeng Zhang, Tongyan Zheng, Yunxia Liu, Luping Dai, Yi Xie, Xianchao Shang, Li Zhang, Long Yang, Ling Yuan, Xin Hou

**Affiliations:** 1 College of Plant Protection, Shandong Agricultural University 34734, Tai'an, China; 2 Department of Plant and Soil Sciences, Kentucky Tobacco Research and Development Center, University of Kentucky 4530, Lexington, Kentucky, USA; Luonnonvarakeskus, Oulu, Finland

**Keywords:** rotation, tobacco, woad, microbial community, species diversity

## Abstract

Continuous tobacco monocropping leads to soil degradation and yield reduction. To address this, we evaluated the effects of tobacco (*Nicotiana tabacum* L.)-woad (*Isatis tinctoria* L.) rotation (A2, A4) compared to tobacco monoculture (A1) and woad monoculture (A3) on soil health and crop quality over a multi-year period. Methods involved comparative analysis of soil nutrients, enzyme activities, microbial community structure, and crop chemical composition and economic value. Key results demonstrated that tobacco-woad rotation significantly improved soil fertility. The tobacco-woad rotation could increase the content of organic matter, alkaline available nitrogen, available phosphorus, and available potassium in the soil, which were increased by 1.44%, 17.96%, 4.61%, and 16.20%, respectively, compared to tobacco monoculture. Soil urease and catalase activities, particularly urease (increased by 2.31 times), were significantly enhanced during the tobacco pre-growth period under rotation. Soil microbial communities were significantly restructured under tobacco-woad rotation versus monocropping. Bacterial phyla Acidobacteria, Gemmatimonadota, and Methylomirabilota were enriched in tobacco-woad rotation (A2) relative to tobacco monoculture (A1), while Chloroflexi, Methylomirabilota, and Verrucomicrobiota increased in woad-tobacco rotation (A4) versus woad monoculture (A3). Fungal shifts featured decreased Ascomycota and Basidiomycota with increased Mortierellomycota in both rotations, alongside reduced Chytridiomycota in A4. Rotation enriched key bacterial genera (*MND1*, *Nitrospira*, *Subgroup-10*, and *RB41*) and fungal taxa (*Mortierella*, *Saccharomyces*, and *Saitozyma*). Crucially, rotation harmoniously improved the chemical composition of both tobacco and woad leaves, increasing reducing sugars, total sugars, nicotine, potassium, and the sugar ratio in tobacco. The proportion of high-quality tobacco leaves post-curing increased by 10.24% (A2), contributing to a significantly higher total crop production value. In conclusion, tobacco-woad rotation effectively alleviates soil degradation associated with continuous tobacco cropping by enhancing soil nutrient availability, boosting key enzyme activities, and optimizing the structure and interactions of the soil microbial community. These soil improvements collectively drive superior crop quality and economic returns, supporting their adoption as a sustainable agricultural practice.

**IMPORTANCE:**

(i) The effects of rotation of tobacco with woad on the quality of tobacco production were clarified using physiological and biochemical analyses. (ii) The effects of rotating tobacco with woad on soil microorganisms were revealed by microbiome sequencing of tobacco soils. Tobacco-woad rotation significantly improved the relative abundance of soil-dominant bacteria and decreased the relative abundance of harmful fungi. (iii) An efficient cultivation model of tobacco and woad suitable for Shandong was established by combining soil microbiomics with tobacco plant growth and development. Rotation of tobacco to woad gave the best results.

## INTRODUCTION

Tobacco is an extremely crucial cash crop and the main source of tax revenue in China. As the quality of human life increases, activities such as urban and rural construction and industrial development are expanding, resulting in a continuous decrease in the amount of land available for cultivation and a decline in species diversity (1). Undoubtedly, successive years of cultivation have caused many problems in production, such as low yields, soil nutrient imbalance, and the emergence of diseases, which have wreaked havoc on agricultural systems (2, 3). Continuous cropping can lead to soil nutrient imbalance, inhibit tobacco growth, and cause soil-borne pests and diseases. The obstacle of monoculture has become a global problem in tobacco production. Crop rotation is a traditional cropping method that can reduce pressure on the soil and promote adaptation of agricultural structure.

There has been much research about the impact of rotation. Studies have shown that when tobacco is rotated with suitable crops, the previous crop has a positive impact on quality and yield, not only improving the physicochemical properties of the soil but also improving the microenvironment for tobacco growth and effectively controlling continuous cropping disturbances (4). Compared to tobacco monoculture, Yan et al. (5) found that the introduction of soybean into the rotation pattern reduced soil organic matter mineralization, which further benefits carbon sequestration and climate change mitigation (5). A related study showed that wheat-maize rotation with soybean and other crops can reduce soil mineral nitrogen during wheat growth and effectively improve soil quality and wheat yield (6). By analyzing the impact of four legumes in rotation with wheat and maize, Liu et al. (7) found that the introduction of legumes in low area, low input systems would effectively increase the yield potential of the rotation system, increase wheat yields in the later crop while maintaining soil carbon stocks, and improve crop yields through enhanced multifunctionality of the soil ecosystem (7). A study establishing a 4-year rotation confirmed that rotation had a positive effect on biomass yield and N uptake, increasing soil C and N stocks and reducing nitrate leaching (8).

Much work has shown that microorganisms contribute significantly to soil nutrition and fertility (9, 10). Appropriate planting patterns help to maintain the activity of soil microorganisms and increase the diversity of beneficial bacteria, thus modifying the soil environment (11). Cerecetto et al. (12) studied soil physicochemical properties, soil and interroot microbial communities, and aboveground biomass and nutrient content of oats after crop rotation (12). The results showed that beneficial bacterial genera were increased after rotation of pasture with oats, which better maintained soil bulkiness and larger aggregates and favored the growth and development of aboveground parts of the plant. A study of continuous peanut cultivation found that continuous cultivation contributed to the emergence of pathogenic bacteria in the soil that induced peanut root rot, and the incidence of root rot was significantly higher than in rotational cropping (13). Yang et al. (14) reported that soybean-based crop rotation significantly reduced soil acidification and positively influenced soil microbial activities and enzyme activities, contributing to higher crop yields and reduced greenhouse gas emissions. Consequently, soil characteristics and crop species should be considered in crop rotation (14).

Both the roots and leaves of woad, a genus in the cruciferous family, are used medicinally and are known as Panax and Daphyllum, respectively. Early studies have confirmed that rotating woad with mixed beans and mung beans can reduce the incidence of woad diseases while increasing yield (15). However, comparative studies elucidating how tobacco monoculture (A1), woad monoculture (A3), and distinct tobacco/woad rotations (A2/A4) differentially influence soil microbial communities and their functional linkages to soil health remain lacking. We specifically hypothesized that (i) tobacco-woad rotations would significantly enhance soil nutrient availability compared to monocultures; (ii) rotation regimes would restructure microbial communities by enriching taxa associated with nutrient cycling and soil health; and (iii) shifts in key microbial populations would directly correlate with improved soil nutrient status. This study provides mechanistic insights into how tobacco-woad rotation mitigates monoculture degradation by linking microbial ecology with soil functionality.

## MATERIALS AND METHODS

### Experimental treatments and sampling

The trials were conducted in Qingya Village, Shijiaheqiaogou Village (36.31°N, 118.41°E), Sitou Town, Weifang City, Shandong Province, China, and the Tobacco Comprehensive Laboratory, Shandong Agricultural University in 2023. At the beginning, the soil properties (5–15 cm) were as follows: 7.39 pH, 16.31 g kg^−1^ organic matter, 48.07 mg kg^−1^ total N, 9.97 mg kg^−1^ available P, and 112.4 mg kg^−1^ available K. All the fertilizers were applied following the conventional fertilization practice (Table S1).

Our trial was divided into four treatments: (i) Tobacco monocropping; (ii) woad planted in 2022 and tobacco planted in 2023; (iii) woad monoculture; and (iv) tobacco was planted in 2022 and woad was planted in 2023 (Table 1).

**TABLE 1:** Tobacco and woad rotation planting pattern^*a*^

Treatments	Planting patterns
A1	Tobacco monoculture (110 cm row spacing and 50 cm plant spacing between two tobacco plants, planting 1,200 tobacco per acre)
A2	Woad planted in 2022 and tobacco planted in 2023 (110 cm row spacing and 50 cm plant spacing between two tobacco plants, 20 cm row spacing between two rows of woad, and 25 cm plant spacing)
A3	Woad monoculture (20 cm row spacing between two rows of woad and 25 cm plant spacing)
A4	Tobacco was planted in 2022 and woad was planted in 2023 (110 cm row spacing and 50 cm plant spacing between two tobacco plants, 20 cm row spacing between two rows of woad, and 25 cm plant spacing)

^*a*^ Planting system of tobacco and woad rotation. A1, tobacco monoculture; A2, woad-tobacco rotation; A3, woad monoculture; A4, tobacco-woad rotation. Row spacing: Refers to the horizontal distance between the center lines of adjacent rows of crops. It measures the width between rows. Plant spacing: Refers to the horizontal distance between the center points of adjacent plants in the same row. It measures the distance between plants in the same row.

Planting system of tobacco and woad rotation. A1, tobacco monoculture; A2, woad-tobacco rotation; A3, woad monoculture; A4, tobacco-woad rotation. Row spacing: Refers to the horizontal distance between the center lines of adjacent rows of crops. It measures the width between rows. Plant spacing: Refers to the horizontal distance between the center points of adjacent plants in the same row. It measures the distance between plants in the same row.

On 10 September 2023, soil samples were taken at tobacco maturity using the five-point sampling method. Soil samples were taken between the rows of tobacco or woad, with five soil cores (5–15 cm) taken from each experimental area. The five soil samples from each plot were combined to create a composite sample. Each treatment was replicated three times, and approximately 100 g of soil was taken for each replicate, mixed thoroughly to filter out obvious impurities, and grouped into A1, A2, A3, and A4. Samples were sent to the laboratory immediately after collection. The retrieved samples were stored separately, partly naturally dried for 1 week, and then passed through a 1 mm sieve for soil nutrient determination. Part of the samples were refrigerated at 4°C for enzyme activity determination, and the rest were frozen at −80°C for 16S rRNA gene amplicon sequencing.

### Soil nutrient analyses and enzyme activities

Soil pH was determined using a pH meter (air-dried soil/water, 1/2.5, wt/wt). The alkaline hydrolysis diffusion method was used to determine soil alkaline nitrogen (AN) (16). Soil alkaline phosphorus (AP) was determined by NaHCO_3_ molybdenum antimony colorimetry, and soil available potassium (AK) by NH_4_OAc flame photometry (17). The dichromate volumetric method was used to measure total soil organic carbon (SOC) (18, 19). Soil sucrase activity was determined using the 3,5-dinitrosalicylic acid colorimetric method (20); urease activity was determined using the sodium phenolate-sodium hypochlorite colorimetric method (21); catalase activity was determined using the potassium permanganate titration method; β-glucosidase activity was determined using the p-nitrophenol colorimetric method (22). Enzyme activities were measured using an ultraviolet spectrophotometer (Hitachi UV1900).

### Soil DNA extraction, PCR amplification, and sequencing

Microbial DNA was extracted from 0.25 g of soil using the DNeasy PowerSoil Pro Kit with triplicate technical replicates pooled. All extracts met quality thresholds (≥10 ng/µL DNA, A260/A280 = 1.8–2.0) prior to amplification. The microplate reader (Gene Company Limited, Synergy HTX) was used to determine DNA concentration and purity. Amplification was performed according to the assay, and the integrity of PCR products was checked by agarose electrophoresis at 1.8% concentration (Bomei Fuxin Technology Co., Ltd., Beijing, China).

Amplification targeted the bacterial 16S rRNA gene and fungal ITS rRNA gene using genomic DNA as template. Bacteria-specific fragments were highly variable 16S rRNA gene V3-V4 regions with the 338F: 5′-ACTCCTACGGAGGCAGCA-3′/806R: 5′-GGACTACHVGGTW-TCTAAT-3′ primer sets for bacteria. Fungi-specific fragments were ITS rRNA gene ITS1 regions using the ITS1F: 5′-CTTGGTCATTTAGAGGAAGTAA-3′/ITS2: 5′-GCTGCGTTCTTCATCGATGC-3′ primer sets for fungi. All amplifications were performed in 10 µL reactions with 0.3 µL of each primer, 0.2 µL KOD FX Neo, 2 µL 2 mM dNTPs, 5 ng template, 0.5 µL KOD FX Neo Buffer, and ddH_2_O to make up to 10 µL. PCR conditions for the 16S V3-V4 rRNA gene were an initial denaturation step at 95°C for 5 minutes, followed by 25 cycles of 95°C for 30 seconds, 50°C for 30 seconds, and 72°C for 40 seconds, and a final extension step at 72°C for 7 minutes. Target regional PCR products were purified and analyzed on 1.8% agarose gels. Results were quantified using Image J software, 150 ng of each sample was mixed and pooled, and the mixed samples were purified using e.Z.N.A.TM Cycle-Pure Kit (omega) columns prior to gel cutting and recovered by gel cutting using the Monarch DNA Gel Recovery Kit. Finally, the paired-end sequencing was performed on the Illumina NovaSeq 6000 platform at Biomarker Technologies Co., LTD (Beijing, China). Raw reads were trimmed using Trimmomatic v0.33, followed by primer removal with cutadapt v1.9.1 (error rate = 0.1), generating clean reads. Clean reads were merged via USEARCH v10 with subsequent length filtration (400–550 bp for 16S; 200–600 bp for ITS). Chimeric sequences were detected and eliminated using UCHIME v4.2 to obtain effective reads for downstream analysis.

### Quality and production value measurement

The tobacco yield of each treatment was counted after harvesting and roasting, and the woad yield of A2, A3, and A4 was counted. The output value of the crop was calculated based on the yield of tobacco and woad according to the prices of the nearby market. Finally, the total production value of the tobacco fields of each treatment was determined. Representative samples of tobacco after roasting (B2F, X2F) were selected for the determination of reducing nicotine, total nitrogen, sugars, and total sugars content using a continuous flow analyzer in accordance with the tobacco industry standard YC/T159-161-2002 Continuous Flow Method for the Chemical Composition of Tobacco and Tobacco Products. The potassium content of tobacco was determined using atomic absorption spectrometry.

### Bioinformatics and statistical analyses

Microsoft Excel 2019 and Origin 2022 were used to process the soil nutrient and soil enzyme activity to obtain trend lines and error values for the raw data and graphs, and the data were analyzed for significance using the SPSS Statistics 26.0 method.

**Fig 1:**
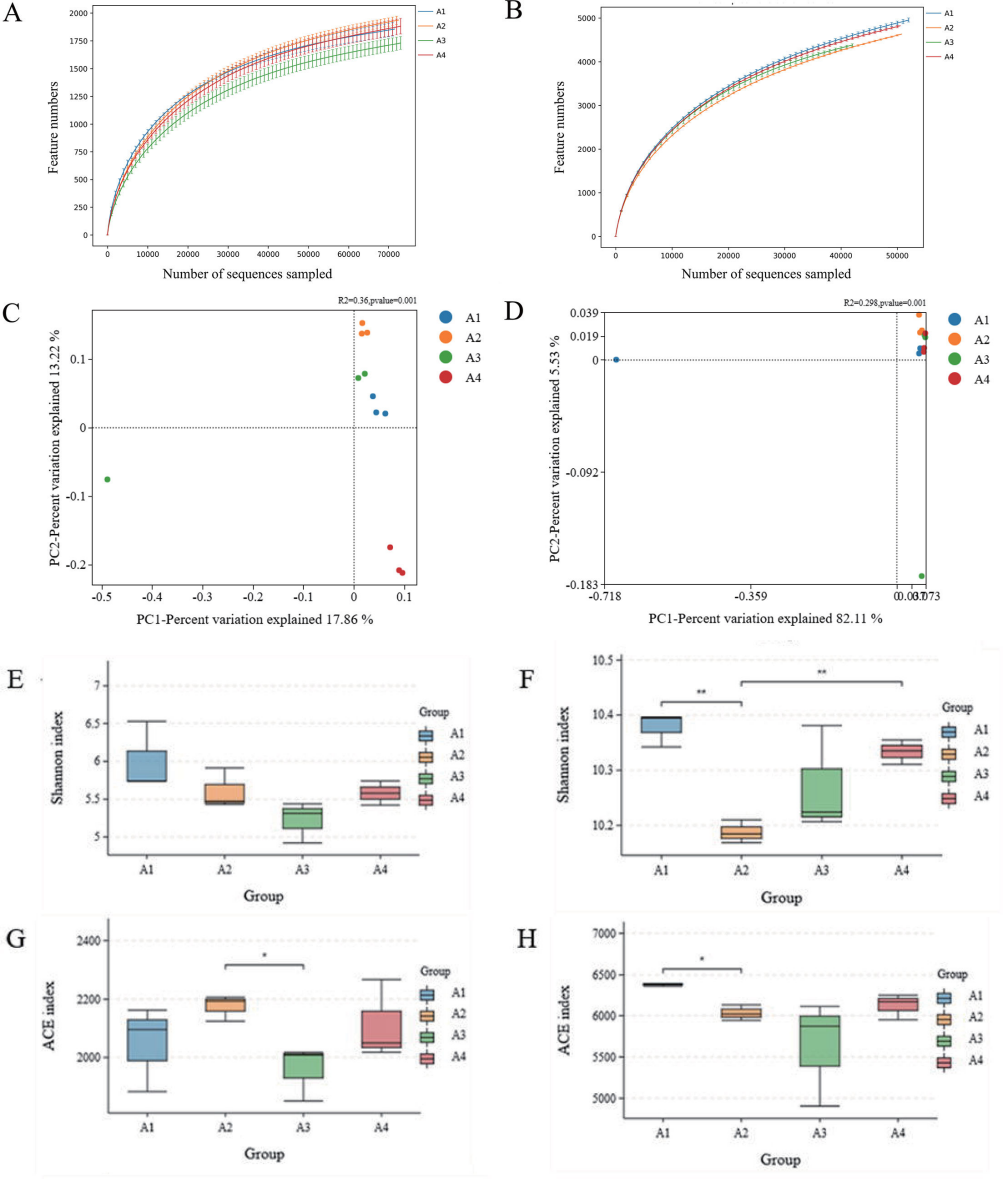
Multi-sample rarefaction curves displaying the availability of bacterial (16S) (**A**) and fungal (ITS) (**B**) samples under A1, A2, A3, and A4. PCoA and Bray-Curtis distances indicate similarity between different treatments of bacteria (**C**) and fungi (**D**). After normalization to even sequencing depth, Shannon reflects the diversity of different processing bacterial (**E**) and fungal (**F**) at the genus level, and ACE reflects the dominance of different processing bacterial (**G**) and fungal (**H**) at the genus level. Each symbol represents an independent biological replicate (*n* = 5 per treatment group). The three marked points shown in the figure are group centroids, which are used to highlight differences between treatments. Asterisks indicate significant differences according to Student’s t-test (*P < 0.05, **P < 0.01). A1, tobacco monocropping; A2, woad-tobacco rotation; A3, woad monocropping; and A4, tobacco-woad rotation.

Quality control was performed on the raw sequenced sequences, including filtering for low quality and filtering for length. The resulting high-quality sequences were clustered into operational taxonomic units (OTUs) at a 97% nucleotide identity threshold using UPARSE, with chimeric sequences removed via UCHIME. Taxonomic annotation was performed against the SILVA 138 database using a confidence cutoff of 0.8, assigning features based on sequence composition. Based on the results of the feature analysis, the samples were taxonomically analyzed at each taxonomic level to generate a community structure diagram and a species clustering heat map for each sample at the phylum and genus taxonomic level. Each treatment group was set up with five independent biological replicates (i.e., five independent experimental fields). Before DNA extraction, each soil sample was divided into three technical replicate subsamples for parallel extraction, which were then combined to construct the library. Species diversity within each sample was analyzed by alpha diversity, and the abundance (ACE index) (23) and diversity (Shannon index) of microbial communities (24) were calculated in phyloseq v1.38.0 (25). Sequencing depth was downsampled to the minimum value the rrarefy() function in the R vegan package (v2.5-7) under R software (version 3.6.1) (26), and the downsampling curve confirmed that the subsampling depth was sufficient to capture microbial diversity (Fig. 1A and B). All subsequent diversity calculations were performed using standardized OTUs. The similarities and differences in bacterial and fungal community composition between the four planting methods were investigated and presented using Principal Coordinate Analysis (PCoA) based on Bray-Curtis distance, implemented with the ape::pcoa() function (27). Permutational multivariate analysis of variance (PERMANOVA) was used to further test whether the four groups of bacterial and fungal communities were significantly different. In this study, network analysis based on Spearman’s rank analysis was performed using the 50 most abundant phyla in the microbial community to explore the relationships between different species in multiple samples. The correlation between the soil bacterial communities was concluded by a direct, strong, and significant correlation (*ρ* > 0.6, *P* < 0.01) between their interrelationships. The correlation between microbial communities and soil environmental parameters was investigated using redundancy analysis (RDA).

## RESULTS

### Effect of rotation on the soil nutrients and enzyme activities

The nutrient characteristics of the different soil samples are described in Table 2. Obviously, significant differences in soil physico-chemical characteristics were found between A1, A2, A3, and A4 treatments. Soil pH decreased significantly by 8.3% in A4 compared to A3. In particular, A2 significantly increased soil SOC content from 12.97 to 14.79 g/kg. In addition, A2 treatment significantly increased AK from 118.62 to 144.77 mg/kg. It is worth noting that soil AN and AP contents were not significantly increased in the rotation treatments.

**TABLE 2:** Effect of rotation on the soil nutrients^*a*^

Treatment	pH	SOC (g/kg)	AN (mg/kg)	AP (mg/kg)	AK (mg/kg)
A1	7.39 ± 0.05c	12.97 ± 0.39b	17.73 ± 0.81a	9.44 ± 0.94a	118.62 ± 0.4bc
A2	7.73 ± 0.04b	14.79 ± 0.46a	18.2 ± 0.61a	6.72 ± 0.72b	144.77 ± 0.6ab
A3	7.98 ± 0.16a	14.19 ± 0.53a	14.47 ± 0.53b	6.37 ± 0.37b	169.57 ± 9.24a
A4	7.32 ± 0.07c	12.59 ± 0.57b	15.63 ± 1.13b	6.6 ± 0.09b	106.75 ± 0.26c

^*a*^ Different letters indicate the significant differences between treatments according to Duncan’s multiple range test (DMRT) at *P* < 0.05. Numbers following plus or minus signs represent standard deviations. A1, tobacco monocropping; A2, woad-tobacco rotation; A3, woad monocropping; A4, tobacco-woad rotation; SOC, soil organic carbon; AN, alkaline nitrogen; AP, available phosphorus; AK, available potassium.

As shown in Table 3, A2 and A4 had obviously higher levels of soil enzyme activities than A1 and A3. Although the soil sucrase activity decreased significantly from 21.88 to 13.7 U/g in the A2 treatment (*P* < 0.05). However, soil sucrase activity was significantly higher in the A4 treatment than in the A3 treatment. In A2 treatments, urease activity was found to increase from 947.33 to 3138.17 U/g compared to A1. The activity of soil catalase in A2 was significantly increased from 1.48 to 5.1 U/g compared to that in A1. Furthermore, the activity of soil β-glucosidase was significantly increased on A4 treatments compared to A3.

**TABLE 3:** Effect of rotation on the soil enzyme activities^*a*^

Treatment	Sucrase (U/g)	Urease (U/g)	Catalase (U/g)	β-glucosidase (U/g)
A1	21.88 ± 0.88a	947.33 ± 22.9d	1.48 ± 0.04c	12.1 ± 0.66a
A2	13.7 ± 0.4b	3138.17 ± 7.63b	5.1 ± 0.05a	4.03 ± 0.8c
A3	9.89 ± 0.18c	3173.79 ± 11.66a	1.85 ± 0.06b	4.7 ± 1.54c
A4	14.32 ± 0.45b	1354.45 ± 8.81c	1.53 ± 0.51c	9.23 ± 0.45b

^*a*^ Different letters indicated the significant differences between treatments according to DMRT at *P* < 0.05.

### Effect of rotation on soil bacterial and fungal community diversity and structure

Microbial DNA extracted from differentially treated soils underwent high-throughput sequencing. Sequences were clustered into OTUs using a 97% nucleotide similarity threshold. The rarefaction curves had clear asymptotes, an indication that sampling was almost complete (Fig. 1A and B). The results showed that there were a total of 7,318 bacterial OTUs in soil samples from A1, 7,017 bacterial OTUs in soil samples from A2, 7,668 bacterial OTUs in soil samples from A3, and 7,334 bacterial OTUs in soil samples from A4. Shannon and ACE diversity indices of soil bacterial communities were not significantly different between treatments (Fig. 1E and F). There were a total of 3,231 fungal OTUs in the A1 soil samples and 3,477 fungal OTUs in the A2 soil samples, which were not significantly different. The Shannon index and the ACE diversity index of the soil fungal communities were significantly different between A1 and A2 (Fig. 1G and H).

PCoA showed the overall similarity of the bacterial and fungal community structure across the different soil groups by the different OTUs of the sequencing results. PCo1 accounted for 17.86% of the change in bacterial community composition, while PCo2 accounted for 13.22% (Fig. 1C). PCo1 accounted for 82.11% of the change in fungal community composition, whereas PCo2 accounted for 5.53% (Fig. 1D). PCoA ordination based on Bray-Curtis distances demonstrated significant divergence in soil microbial communities under different rotation regimes. PERMANOVA provided even stronger evidence that the composition of the microbial community was significantly variable depending on the cropping pattern. Rotations explained 36% of the variance in bacterial communities (*R²* =0.36, *P* < 0.001). The distribution of centroids between different treatments in PCoA showed significant separation, confirming rotations drive bacterial succession through nutrient availability (Fig. 1C). Rotations explained 29.8% of the variance in fungal community (*R²* =0.298, *P* < 0.001), but its lower explanatory power and PCoA overlap suggest stronger constraints from host specificity or legacy effects (Fig. 1D). This indicates that optimizing crop rotation design has greater potential for improving soil function mediated by bacteria.

### Effect of rotation on soil bacterial and fungal community composition

**Fig 2:**
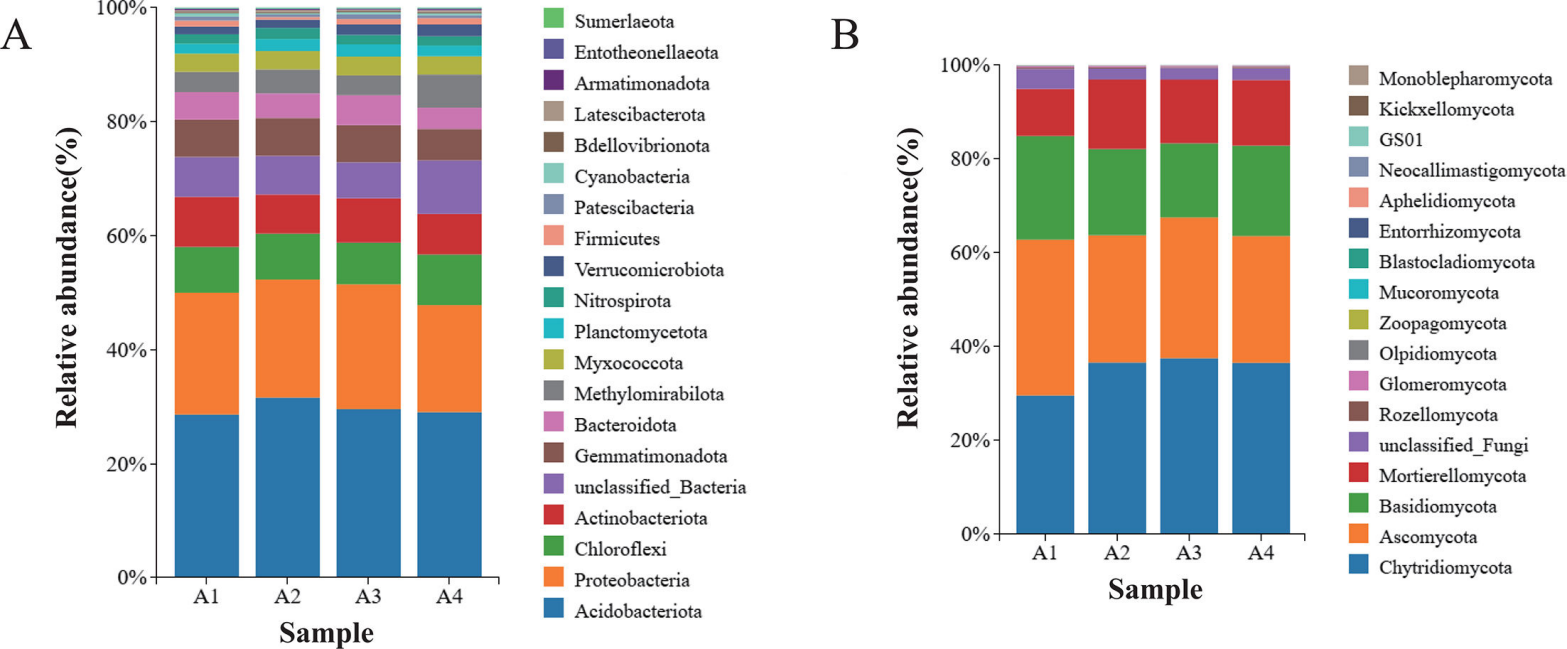
Composition of bacterial community (**A**) and fungal community (**B**) in soil at the phylum level.

**Fig 3:**
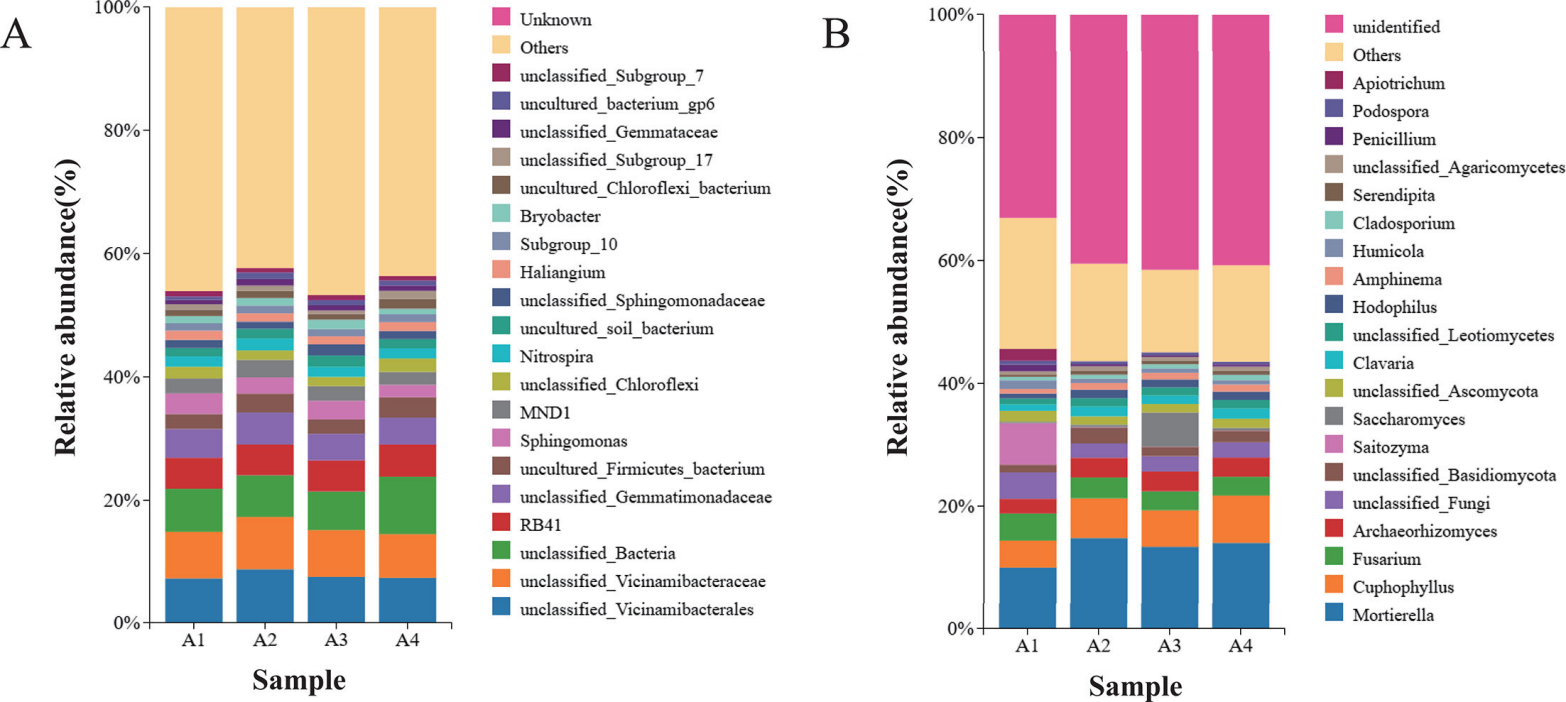
Composition of bacterial community (**A**) and fungal community (**B**) in soil at the genus level.

As shown in the species distribution stacked bar plot (Fig. 2 and 3), tobacco-woad rotation significantly restructured soil microbial community composition and altered the relative abundance of dominant bacterial and fungal phyla and genera. The bacteria of the soil samples at the phylum level are shown in Fig. 2A. Acidobacteria (29%–32%) were the phylum with the highest relative abundance, followed by Proteobacteria (19%–22%), Chloroflexi (7%–9%), Actinobacteriota (7%–9%), and Gemmatimonadota (6%–7%). In particular, Acidobacteria, Gemmatimonadota, Methylomirabilota, Planctomycetota, and Nitrospirae were significantly more abundant in A2 than in A1. In addition, Chloroflexi, Methylomirabilota, and Verrucomicrobiota were significantly more abundant in A4 than in A3. Chytridiomycota (30%–38%), Ascomycota (27%–33%), Basidiomycota (16%–22%), and Mortierellomycota (10%–15%) were the fungi with the highest relative abundance at the phylum level. Compared to A1, Ascomycota and Basidiomycota were less abundant in A2, whereas Mortierellomycota were more abundant in A2. Compared to A3, Chytridiomycota and Ascomycota were less abundant in A4, whereas Mortierellomycota were more abundant in A4 (Fig. 2B).

In addition, a comparison of the soil bacterial and fungal communities between the different treatments was carried out at the genus level (Fig. 2B). Bacterial genera *RB41* (5%), *Sphingomonas* (2%–3%), *MND1* (2%–3%), *Nitrospira* (2%), *Haliangium* (1%), and *Subgroup_10* (1%) exhibited the highest relative abundances across treatments at the genus level. *MND1*, *Nitrospira,* and *Subgroup-10* were significantly more abundant in A2 than in A1. *RB41* and *Subgroup_10* were significantly higher in A4 than in A3. The most abundant fungal genera in the different treatments were *Mortierella* (10%–15%), *Fusarium* (3%–4%), *Archaeorhizomyces* (2%–3%), *Saitozyma* (1%–7%), and *Saccharomyces* (1%–6%). The presence of *Mortierella* and *Saccharomyces* was significantly higher in A2 than in A1. Compared to A3, *Mortierella* and *Saitozyma* were significantly higher in A4.

### Relationships between soil nutrients, soil enzyme activities, and soil bacterial and fungal communities

**Fig 4:**
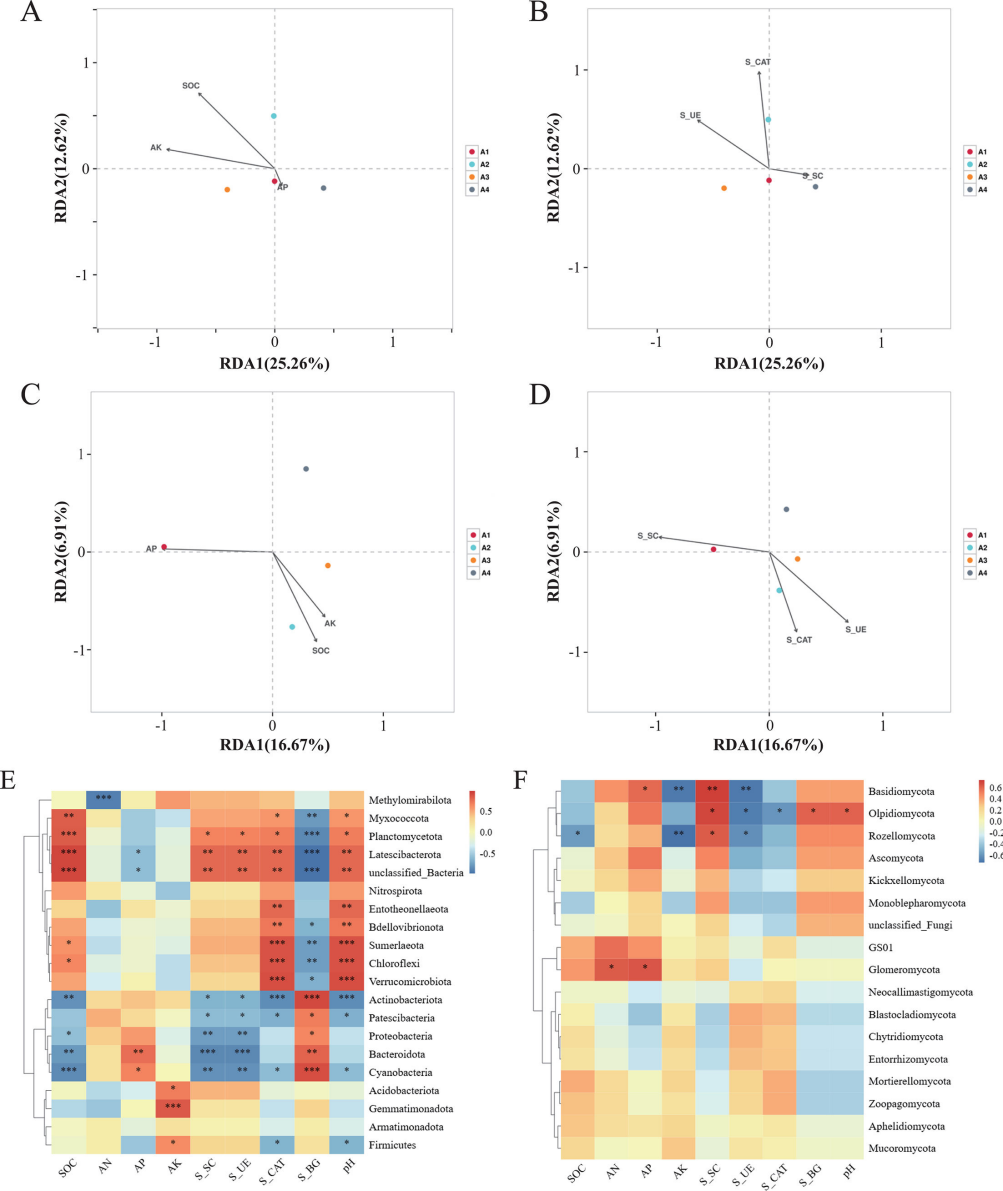
Correlation between bacteria and nutrients (**A**), enzyme activities (**C**) in soil samples and correlation heatmap between nutrients, soil enzyme activities, and dominant bacteria in samples (**E**) correlation between fungi and nutrients (**B**), enzyme activities (**D**) in soil samples and correlation heatmap between nutrients, soil enzyme activities, and dominant fungi in samples (**F**). S_SC, Sucrase; S_UE, Urease; S_CAT, Catalase, S_BG, β-glucosidase. *0.01 < *P* ≤ 0.05, **0.001 < *P* ≤ 0.01, ***P ≤ 0.001.

The influence of soil nutrients and pathogen activity on soil microorganisms was analyzed by RDA. As shown in the figure, it was clear that there were significant differences in the composition of bacteria and fungi under different treatments. SOC, AP, and AK significantly shaped the composition and diversity of soil microbial communities within the rotation system. AP was positively correlated with changes in bacterial communities in A1 and A4 and negatively correlated with A2. SOC and AK were positively correlated with changes in the bacterial communities in A2 and A3 and negatively correlated with changes in A4 (Fig. 4A). AP was positively correlated with changes in fungal communities in A1 and negatively correlated with A2, A3, and A4. SOC and AK were positively correlated with changes in fungal communities in A2 and A3 treatments and negatively correlated with A1 and A4 (Fig. 4C). Regarding the soil enzyme activities of the different treatments, sucrase activities were positively correlated with changes in bacterial communities in A1 and A4 and negatively correlated with A2 and A3. Urease activities were positively correlated with bacterial community changes in A2 and A3 and negatively correlated with A1 and A4 (Fig. 4B). Catalase activities were positively correlated with changes in bacterial communities in A2 and negatively correlated with A1, A3, and A4. The activities of sucrase were positively correlated with the changes in the fungal communities in the A1 treatments and negatively correlated with A2, A3, and A4. Soil urease and catalase activities were positively correlated with changes in fungal communities in A2 and A3 and negatively correlated with A1 and A4 (Fig. 4C and D).

Planctomycetota was strongly associated with the relative abundances of SOC, AK, S_UE, and S_CAT (Fig. 4E). Chloroflexi displayed a positive correlation with soil SOC and S_CAT. Basidiomycota were negatively correlated with AK and S_UE. Olpidiomycota showed a positive correlation with S_SC and S_BG, and Glomeromycota was positively correlated with AN and AK (Fig. 4F).

### Effect of rotation on yield and production value

As shown in Table 4, the output, average price, and output value of tobacco were significantly higher after rotation with woad than after monoculture with tobacco. After rotation with woad, the average output of A2 was 175.47 kg, which increased by 4.07%; the average price of A2 was 29.08 yuan, which increased by 4.23%; the output value benefit of A2 was 5102.67 yuan/667 m^2^, which increased by 8.48%. Similarly, the output, average price, and production value benefit of woad after rotation with tobacco were significantly higher than those of woad monoculture. After rotation with tobacco, the average yield of A4 was 42.67 kg, and the output value benefit was 512.04 yuan/667 m^2^, an increase of 15.45% compared to A3.

**TABLE 4:** Effect of rotation on the output value of tobacco and woad^*a*^

Treatment	Crop	Output (kg/667 m^2^)	Average price (RMB/kg)	Output value benefit (RMB/667 m^2^)
A1	Tobacco	168.60b	27.90b	4703.94b
A2	Tobacco	175.47a	29.08a	5102.67a
A3	Woad	36.96b	12.00a	443.52b
A4	Woad	42.67a	12.00a	512.04a

^*a*^ Data within the same crop column followed by different letters are significantly different (*P* < 0.05).

### Effect of rotation on quality

The tobacco-woad rotation treatments showed a significant increase in the proportion of superior smoke after roasting. The lowest proportion of superior tobacco was found in A1 with 48.34%. A2 had 53.29%, which was 10.24% higher than A1. The results showed that rotating the tobacco-woad could increase the proportion of superior tobacco after roasting and effectively improve the tobacco quality.

As shown in the Table 5, the chemical composition of tobacco leaves in the upper leaves (B2F) of all treatments was within the acceptable range, and the differences between monoculture and rotation treatments were significant. The contents of reducing sugars, total sugars, nicotine, potassium, and the ratio of two sugars were increased in the upper leaves of all the treatments after the rotation of the tobacco crop. And the contents of total sugars, nicotine, potassium, and the nitrogen-alkali ratio were increased in the central leaves of all treatments after tobacco-woad rotation. The total sugar contents of the central leaf (C3F) of A1 and A2 treatments were 24.09% and 24.26%, respectively, which was above the suitable range of total sugars (18-24%), and the puff flavor of the tobacco was bland when the total sugar content was too rich. The potassium content of the central leaf of the A1 treatments was slightly below the appropriate range of 2%, while the potassium content of the A2 treatment was 2.03%. The adequate potassium content in A2 treatment contributed to a more balanced sensory profile, characterized by enhanced sweetness and reduced irritation. The sugar-alkali ratio, which is a very important index in evaluating tobacco quality, is in the range of 6–10. The sugar-alkali ratio in the middle leaf of the A1 and A2 treatments was 10.77 and 10.74, respectively, which is outside the appropriate range and will increase the irritation of smoke and flavor. The sugar-alkali ratio in the upper leaf of the rotation treatments was all within the normal range, indicating that tobacco rotation could regulate the sugar-alkali ratio of the tobacco and improve the quality of the tobacco. The nitrogen-alkali ratio is also an important indicator of tobacco quality; its appropriate range is 0.7–1.1. The A4 treatment of the upper leaves was 0.80, which was within the appropriate range. It can be seen that tobacco-woad rotation can improve tobacco quality to a certain extent.

**TABLE 5:** Effects of rotation between tobacco and woad indigotica on the chemical composition of tobacco leaves^*a*^

Treatment	Grade	Reducing sugars (%)	Total sugars (%)	Total nitrogen (%)	Nicotine (%)	K (%)	Glycemic ratio	Total sugar to reducing sugar ratio	Ratio of nitrogen to alkali
A1	B2F	15.54b	21.55b	1.76b	2.27b	1.37b	9.50a	0.72b	0.77a
A2		17.11a	22.20a	2.07a	3.04a	1.69a	7.31b	0.77a	0.68b
A1	C3F	18.11a	24.09b	1.61b	2.24b	1.74b	10.77a	0.75a	0.72b
A2		18.11a	24.26a	1.81a	2.26a	2.03a	10.74a	0.75a	0.80a

^*a*^ Different letters indicated the significant differences between treatments according to DMRT at *P* < 0.05.

And as shown in the Table 6, crop rotation favors the growth and synthesis of active substances in the woad. The uridine, guanosine, (R,S)-gauichine, and adenosine contents in the woad were increased in all the woad treatments after tobacco-woad rotation, which effectively improved the quality of the woad.

**TABLE 6:** Effects of rotation between tobacco and woad indigotica on the chemical composition of woad^*a*^

Treatment	Uridine accumulation (%)	Guanosine accumulation (%)	(R,S)-goitrin accumulation (%)	Adenosine accumulation (%)
A3	0.04b	0.05b	0.10b	0.06b
A4	0.05a	0.07a	0.13a	0.07a

^*a*^ Different letters indicated the significant differences between treatments according to DMRT at *P* < 0.05.

## DISCUSSION

Several studies suggest that different cropping patterns can alter numerous soil properties (6, 28). Tobacco can synthesize more protein, promote cell division and growth, and increase photosynthesis when there is sufficient AN in the soil. As an important indicator, soil AN has a strong influence on the dynamics of the soil nitrogen pool, and its level and dynamics can directly influence soil fertility (29). In this study, soil alkaline nitrogen was not significantly increased in A2 and A4 (Table 2). This may be due to the effect of perennial continuous tobacco cultivation on the uptake of alkaline nitrogen by tobacco, resulting in an imbalance of soil nutrient ratios and soil quality degradation. Soil organic matter content is an important index used to measure soil fertility. In this study, soil organic matter content was significantly higher in A2 (Table 2), indicating that rotation significantly improved soil fertility compared to monoculture. The AK content of A2 was significantly increased compared to A1 (Table 2). Recent studies have also indicated that AK in the soil promotes the synthesis and functioning of carbohydrates, increases the efficiency of light and equipment, facilitates normal plant development, and increases the resistance of plants to pests and drought and promotes nitrogen metabolism in tobacco (30).

The activity of soil enzymes represents the degree of nutrient cycling in the soil (31, 32). The higher the soil sucrase, the higher the soil fertility, because sucrase promotes the accumulation of labile nutrients in the soil environment. Sucrase determines, to a certain extent, the degree of soil maturation and soil fertility, and it determines the intensity of biological activity in the soil (33). Up to the peak of the tobacco crop, sucrase activity in the tobacco rotation was significantly higher than in the continuous crop (Fig. S1). The bacterial assemblages under rotation treatment exhibited positive alignment along the increasing gradient of soil sucrase activity, which was hypothesized to be probably due to woad root secretion influencing soil microbial changes leading to increased soil sucrase activity (Fig. 4B). Soil urease increases the content of nitrogen and carbon compounds in the soil environment and promotes the hydrolysis of urea, the level of which has an important influence on the abundance of microbial organic matter. Urease also represents the level of soil nitrogen and can effectively promote the transformation of SOC and regulate the efficiency of energy metabolism. Similarly, the increase in soil urease activity indicates the improvement of soil fertility, and crop rotation clearly improves soil fertility. Catalase can promote the decomposition of hydrogen peroxide in the soil and reduce its harmful effects on plants. A2 had the highest level of catalase activity, which could explain the greater diversity of microorganisms in crop rotations, in agreement with previous findings (34). The β-glucosidase activity did not change too much throughout the reproductive period of the baked tobacco, but it can be seen that after transplanting to the peak period, the activity of the baked tobacco rotation treatments was higher than that of the monoculture, which played a certain role in improving soil fertility (Fig. S2).

High levels of soil microbial alpha diversity index help to maintain the sustainability and increase the resilience of soil ecosystems and ensure their proper function (35). The Shannon index reflects the diversity of soil microbial communities, and the ACE index is an important indicator of species dominance in soil microbial communities (36). There was a significant difference between A1 and A2 in the ACE diversity index and the Shannon index of the soil fungal communities. Nevertheless, some researchers did not find significant changes in alpha diversity (4), and they did not draw firm conclusions on whether rotation affected the alpha index. No significant differences were found between the Shannon and ACE indices of the bacterial community of two cropping systems (Fig. 1E through H), which is consistent with their findings.

Tobacco-woad rotation significantly restructured soil microbial assemblages, with profound implications for soil health and pathogen suppression. The increase in soil microbial relative abundance may be the result of enhanced metabolic activity and proliferation of key functional groups, which is mainly driven by root exudates from herbaceous plants. Woad is a Chinese herb that can secrete nutrients and exudates when rotated with tobacco, which can affect the microbial community of A2 and A4. The increase in bacterial OTUs for A4 in comparison to monoculture seems to be an indication of this. Tobacco-woad rotation reshaped bacterial communities by enriching functional guilds (Fig. 2): Acidobacteriota dominance correlated with SOC accumulation, indicating enhanced carbon stabilization. *Nitrospira* doubled in relative abundance, driving nitrification efficiency through reduced fungal competition. Actinobacteria can enhance host nutrient acquisition and are well known for their beneficial effects on plants (37, 38). In addition, Gemmatimonadota may be able to reduce the proportion of harmful fungi (39). Rotation optimizes bacterial functional traits for nutrient retention and greenhouse gas mitigation. All rotation treatments significantly reduced the relative abundance of Ascomycota and Basidiomycota taxa (Fig. 3)—phyla harboring pathogens like *Fusarium*—while elevating the relative abundance of Mortierellomycota, with *Mortierella* becoming the dominant genus. This shift correlates with woad-derived glucosinolates inhibiting *Fusarium* growth and *Mortierella*’s phosphorus-solubilizing capacity. The synergy between *Saitozyma* and *Nitrospira* enhanced nitrogen cycling. This suggests that roasted tobacco may increase the relative abundance of beneficial and decrease the relative abundance of harmful soil microorganisms and regulate the balance of soil microbial populations after rotation with woad.

Numerous studies have shown that rotation patterns play an intrinsic role in the composition of microbial communities (12, 13). By combining microbial composition with soil chemical properties through RDA, we found that soil nutrients were significantly associated with changes in soil microbial communities (Figure A, C). In addition, environmental indices such as soil SOC, AP, and AK all accelerated microbial community changes, confirming the findings of Zhang et al. (40).

Indicating the potential role of rotation in crop production, the chemical composition of tobacco leaves was increased in rotation treatments. Since microbiologically rich soils have higher organic matter decomposition and plant productivity, the improvement in tobacco yield and quality in rotation may be related to the ability to regulate the interactions of the belowground parts of the crop (41). Tobacco-woad rotation can increase the relative abundance of beneficial bacteria in the soil, reduce the relative abundance of harmful fungi, accelerate the release of enzymes in the soil, accelerate the decomposition of organic matter, improve the tobacco plant growth environment, and enhance tobacco growth and development.

### Conclusion

This study demonstrates that tobacco-woad rotation significantly improves soil health and crop productivity through targeted restructuring of soil microbial communities. Rotation consistently enhanced key fertility indices (SOC, AN, and AK) compared to monoculture systems, establishing a foundation for improved nutrient cycling. Compared to monoculture, rotation enriched beneficial microorganisms, including Acidobacteria, Nitrospirae, and Mortierellomycota, while suppressing potential risk fungi (Ascomycota and Basidiomycota). These microbial shifts were significantly correlated with improvements in key soil nutrients (SOC, AK, and AN), demonstrating a mechanistic link between rotation-induced community restructuring and enhanced soil functionality. Consequently, tobacco-woad rotation optimizes the soil microbial community structure, leading to measurable improvements in soil enzyme activities, tobacco leaf quality, and overall crop yield and economic return. Future research should focus on elucidating the specific functional mechanisms by which woad cultivation drives these beneficial microbial shifts.

## Data Availability

The raw sequencing data generated in this study have been deposited in the NCBI Sequence Read Archive (SRA) under accession number PRJNA1331954. The original contributions presented in the study are included in the article/supplementary material. Further inquiries can be directed to the corresponding authors.

## SUPPLEMENTAL MATERIAL

Supplemental material spectrum.03016-24-s0001.docx Table S1; Fig. S1 and S2.

ASM does not own the copyrights to Supplemental Material that may be linked to, or accessed through, an article. The authors have granted ASM a non-exclusive, world-wide license to publish the Supplemental Material files. Please contact the corresponding author directly for reuse.
