# Supplementary material for: Rotation-driven changes in physicochemical properties modulate soil microbial diversity and community complexity in tobacco-woad soils
Source: Microbiol Spectr. 2025 Nov 17;14(1):e03016-24. doi: 10.1128/spectrum.03016-24 (PMC12772243; doi:10.1128/spectrum.03016-24)
Supplement: Supplemental material — Table S1; Fig. S1 and S2. [file spectrum.03016-24-s0001.docx]

**Table S1** | Baseline physicochemical properties and historical management practices of the experimental site prior to trial initiation

| Soil Property | Mean ± SD | Practice | Specification |
| --- | --- | --- | --- |
| pH | 7.39±0.12 | Prior crop | Continuous tobacco monoculture |
| SOC (g/kg) | 16.31±0.54 | Fertilization | 150-75-100 kg NPK ha⁻¹ yr⁻¹ |
| AN (mg/kg) | 48.07±1.23 | Tillage | Rotary tillage (20 cm depth) |
| AP (mg/kg) | 9.97±0.41 | Irrigation | Drip irrigation (seasonal 350 mm) |
| AK (mg/kg) | 112.4±3.2 | Pesticide application | Chlorpyrifos 0.5 kg ha⁻¹ (bi-annual) |


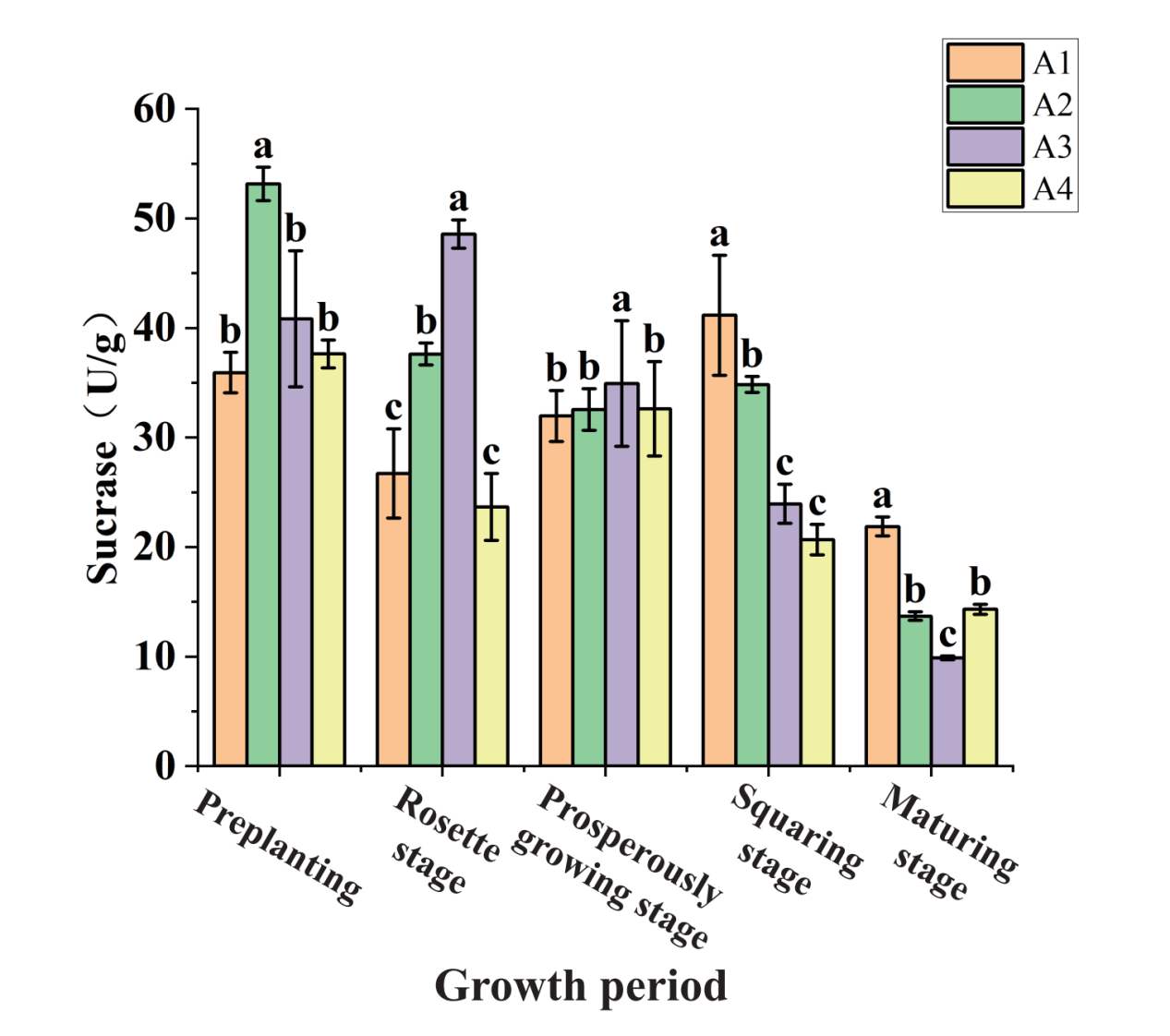


**Figure S1** | Effects of rotation between tobacco and woad on sucrase activity in soil at different periods of time

Different letters indicated the significant differences between treatments according to Duncan’s Multiple Range Test (DMRT) at P < 0.05. Numbers following plus or minus signs represent standard deviations. A1, tobacco monocropping; A2, woad-tobacco rotation; A3, woad monocropping; A4, tobacco-woad rotation. As follows.


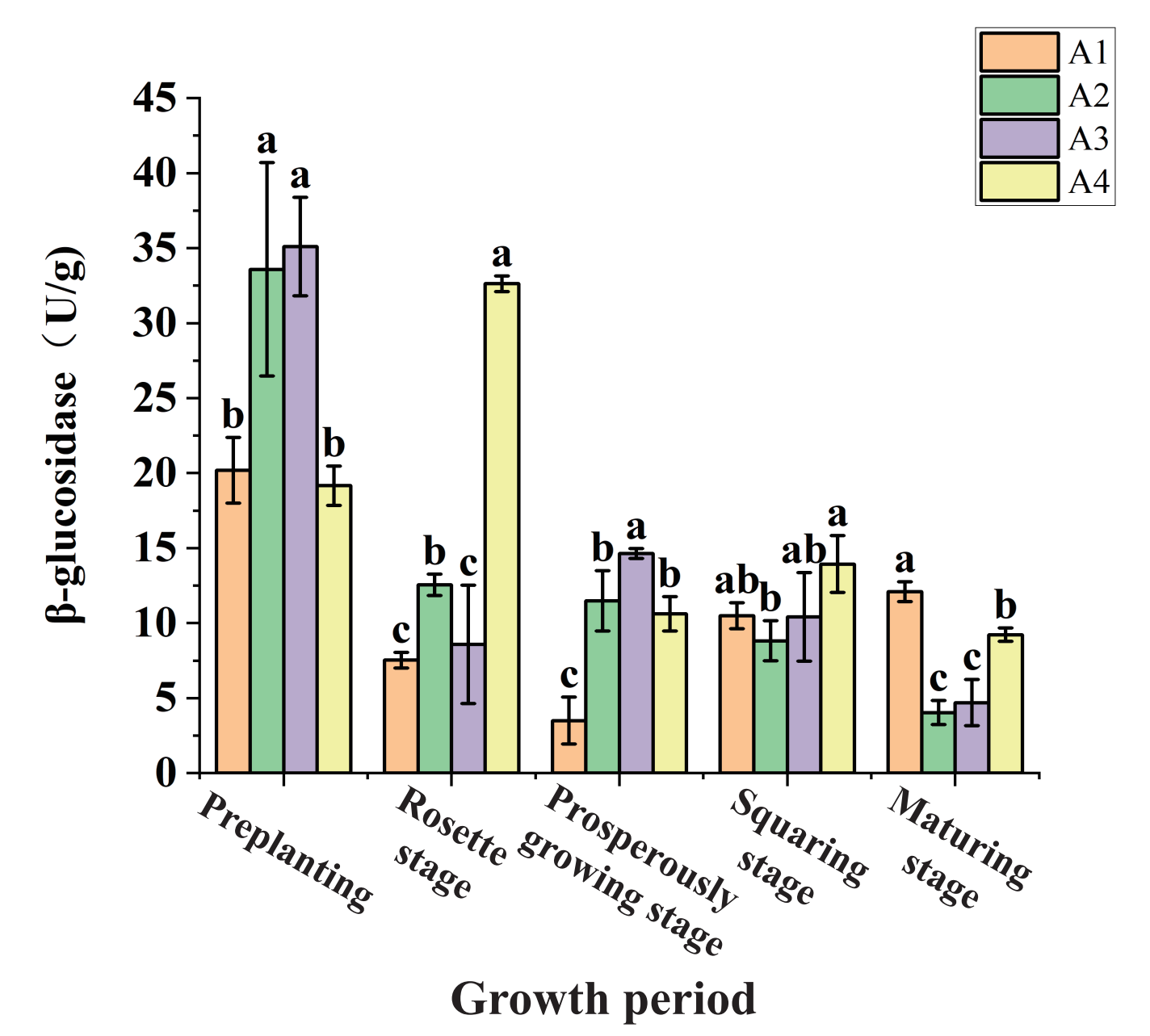


**Figure S2** | Effects of rotation between tobacco and woad on β-glucosidase activity in soil at different periods of time
